# Borderline personality disorder classification based on brain network measures during emotion regulation

**DOI:** 10.1007/s00406-020-01201-3

**Published:** 2020-12-02

**Authors:** Henk Cremers, Linda van Zutphen, Sascha Duken, Gregor Domes, Andreas Sprenger, Lourens Waldorp, Arnoud Arntz

**Affiliations:** 1 Department of Clinical Psychology, University of Amsterdam, Nieuwe Achtergracht 129B, 1001 NK Amsterdam, The Netherlands; 2 Department of Clinical Psychological Science, Maastricht University, Maastricht, The Netherlands; 3 Department of Biological and Clinical Psychology, University of Trier, Trier, Germany; 4 Department of Neurology, University of Lübeck, Lübeck, Germany; 5 Department of Psychological Methods, University of Amsterdam, Amsterdam, The Netherlands

**Keywords:** Borderline personality disorder, Machine learning, Classification, Phasic vs. tonic brain connectivity, Networks analysis, Network measures

## Abstract

Borderline Personality Disorder (BPD) is characterized by an increased emotional sensitivity and dysfunctional capacity to regulate emotions. While amygdala and prefrontal cortex interactions are regarded as the critical neural mechanisms underlying these problems, the empirical evidence hereof is inconsistent. In the current study, we aimed to systematically test different properties of brain connectivity and evaluate the predictive power to detect borderline personality disorder. Patients with borderline personality disorder (*n* = 51), cluster C personality disorder (*n* = 26) and non-patient controls (*n* = 44), performed an fMRI emotion regulation task. Brain network analyses focused on two properties of task-related connectivity: phasic refers to task-event dependent changes in connectivity, while tonic was defined as task-stable background connectivity. Three different network measures were estimated (strength, local efficiency, and participation coefficient) and entered as separate models in a nested cross-validated linear support vector machine classification analysis. Borderline personality disorder vs. non-patient controls classification showed a balanced accuracy of 55%, which was not significant under a permutation null-model, *p* = 0.23. Exploratory analyses did indicate that the tonic strength model was the highest performing model (balanced accuracy 62%), and the amygdala was one of the most important features. Despite being one of the largest data-sets in the field of BPD fMRI research, the sample size may have been limited for this type of classification analysis. The results and analytic procedures do provide starting points for future research, focusing on network measures of tonic connectivity, and potentially focusing on subgroups of BPD.

**Electronic supplementary material:**

The online version of this article (10.1007/s00406-020-01201-3) contains supplementary material, which is available to authorized users.

## Introduction

Borderline Personality Disorder (BPD) is characterized by a pervasive pattern of instability of interpersonal relationships, self-image, affect, and impulse control [1]. Particularly, an increased sensitivity to emotions and a dysfunctional capacity to regulate emotions is considered to be one of the hallmark features of BPD [2]. Research generally focuses on increased amygdala and reduced prefrontal activity as the neural mechanism underlying these processes [3]. Furthermore, previous research suggests a disruption of functional connectivity between these brain areas (e.g., [4, 5]). While some research findings indeed fit this pattern, the inconsistency of results on this topic is perhaps even more notable [3]. For example, there is a large discrepancy between studies on the involvement of lateral and medial prefrontal regions in BPD during emotion regulation [3]. There are several potential reasons for this, such as small samples (low statistical power) and flexibility in analyses, which we aim to address in the current study.

Graph theory is a powerful framework to evaluate the highly interconnected nature of the brain [6], particularly by addressing the balance between the integration of a network on the one hand, and the segregation into modules on the other [6]. Network measures capture to what extend brain regions form important hubs that connect different submodules of a network [7] and alterations in these hub regions characterize several psychiatric disorders [8]. While network analyses are often based on resting-state functional Magnetic Resonance Imaging (fMRI) data, the principles can be extended to capture task-related network reconfiguration of a network [9]. Here we aim to investigate the predictive power of these different network properties to classify BPD patients.

Predictive modeling through machine learning algorithms [10] provides a framework to quantify how well the different network measures can classify BPD. It can be considered a two-step approach, where the first question is if there is any pattern to detect and classify a target variable. Subsequently, one can aim to assess the contributions (feature weights) of the input data, at least in case of linear classification models, which are most commonly used in fMRI [11]. To date, one study has applied a brain network approach to classifying BPD [12] and displayed a promising classification accuracy of 80%. Increased efficiency of the amygdala, entorhinal cortex, and temporal pole in BPD were some of the main discriminative features, which may reflect clinically well-observed borderline characteristics of emotion processing dysfunction. Yet, this study contained a small sample (20 patients, 10 controls), which necessitates further investigation, and whether or not such models can distinguish BPD from other personality disorders also remains to be investigated. Additionally, this work utilized resting-state connectivity, which does show overlap with task-related activation patterns [13], but does not directly tap into emotion regulation processes.

Here, we investigated whole-brain connectivity during an emotion regulation task, to assess task-state related shifts in network configuration [9, 14]. Specifically, we focused on delineating two characteristics: phasic connectivity: the task-event related changes in brain connectivity (see [9, 15, 16 for comparable approaches] and tonic connectivity: background connectivity of brain regions, unrelated to task-events. We build on our previous work [17, 18] and compare BPD to non-patient controls (NPC) and cluster-C personality disorder patients as a relevant clinical control group. Due to the importance of emotion regulation in BPD, we expected that the phasic connectivity shifts would show the highest classification accuracy. We further examined which brain regions were most important in the classification, particularly the critical functional subnetwork modules related to psychiatric disorders: the emotion (including the amygdala), motivation, cognitive control, and default mode modules of the brain [19]. We hypothesized that BPD would show decreased cognitive control-related phasic connectivity during cognitive emotion regulation compared to controls.

## Materials and methods

A detailed outline of the participant inclusion, the task, as well as fMRI data acquisition and preprocessing is described elsewhere [17, 18]. Below are summaries for each section.

### Participants

Patients with BPD or Cluster-C personality disorders were recruited from mental health clinics at two sites in the Netherlands (Maastricht, Heerlen) and three sites in Germany (Freiburg, Lübeck, Hamburg), see “Brain parcellation and subject inclusion” of the results for the subject inclusion. Non-patient controls (NPC) were recruited from the general population at each site. Participants had to be hetero- or bisexual women aged 18–65 years, and were assessed by trained interviewers according to the DSM-IV criteria using the Structural Clinical Interview (SCID) II and I [20]. All participants provided written informed consent. The study was approved by the local ethical committees.

### Task

**Fig. 1:**
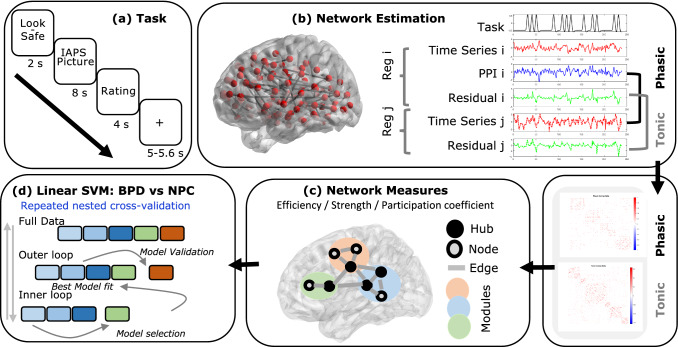
Emotion regulation task and analysis pipeline. **a** Outline of the task: each trial consisted of an instruction (look or safe), IAPS picture presentation, rating period, and fixation-cross screen. **b** Brain regions (Reg) included in the analyses and the statistical models testing the connectivity between two regions (i and j). For each region, the time-series was extracted and multiplied with the task regressors (only one shown), which resulted in the psychophysiological interaction (PPI) terms. The phasic connectivity analyses referred to a regression of the PPI term on the time-series of another region while controlling for the task regressors, time-series, and confounds (not shown). The tonic connectivity analyses referred to the graphical lasso estimation of the residual times series of all regions, after correcting for the task regressors and confounding effects. Each connectivity analysis resulted in a connectivity matrix, which was averaged across runs. **c** Overview of the basic properties of a network consisting of nodes connected by edges and forming subnetworks (modules). Three network measures are used to assess hub functioning: strength, local efficiency, and the participation coefficient. **d** Linear support vector machine classification in a one vs. one fashion (e.g., BPD vs. NPC) using the different network measures from the phasic and tonic connectivity as input features. The different models (e.g., phasic strength, phasic local efficiency, tonic participation coefficient) were assed in a repeated (200 times) nested cross-validation procedure. In the inner loop, the best performing model was selected, re-fitted to the test data of the outer loop, and evaluated on the balanced accuracy of the validation data

Participants performed an adapted version of an emotion regulation paradigm, which involved the presentation of pictures that were preceded by a safe (emotion regulation) or look instruction. During the safe trials, participants were asked to imagine themselves as being in a safe situation. This regulation strategy was based on a central element of schema therapy, one of the main therapies for BPD [17]. There were 4 categories of picture stimuli (negative, neutral, positive, and erotic), and the task consisted of 96 trials divided into 4 runs of 24 trials each (see Fig. 1a for an outline of a trial). As part of the scanning session, participants also underwent resting-state scans before and after the task.

### fMRI data

#### Acquisition and preprocessing

Functional and structural MRI data were acquired with 3 T scanners. The Functional images were acquired with a T2*-weighted echo planar imaging (EPI) sequence, with the following parameters: TR = 2000 ms, TE = 27 ms, flip angle = 90°, FoV = 192 × 192 mm, voxel size = 3 × 3 × 3 mm, and matrix = 64 × 64. In Maastricht 240 images and in Freiburg and Lübeck 252 images were collected. The number of interleaved axial slices in one volume was 32 in Maastricht and 34 in Freiburg and Lübeck. In Maastricht and Freiburg, the T2*-weighted slices were adjusted with a negative tilt of 30°, with the goal of minimizing susceptibility and distortion artifacts within the amygdala. The anatomical images were acquired with a T1-weighted sequence, with the following parameters: TR = 2250 ms, TE = 2.6 ms, flip angle = 9°, Field of View (FoV) = 256 × 256 mm, voxel size 1 × 1 × 1 mm. In total, 192 images were obtained in Maastricht, 160 in Freiburg, and 170 in Lübeck. The preprocessed images from a previous study were used (see [17] for details of the preprocessing pipeline) and transformed from BrainVoyager format into nifti format, using Neuroelf (www.neuroelf.net).

#### Analyses

##### Network estimation

Phasic brain connectivity networks were estimated by performing a whole-brain psychophysiological interaction (PPI) analysis. PPI analyses aim to identify brain regions, where the time-series (physiological signal) connectivity is moderated by a task condition (“the psychological variable”) [21]. Traditionally, this approach has consisted of predefining a source (or seed) region and then estimating its connectivity with other (target) regions. We extended this rationale to a whole-brain network method (see [15, 16] for similar approaches). Here, each brain node is once considered to be the source, while the other nodes are the target. The PPI terms were estimated separately for each task regressor, but since PPI analyses are in general less powerful compared to estimating activation (i.e., the main effect of a task regressor) [22], we opted for a summary model that collapsed the different emotional valences (negative, neutral, positive), and included the general look and safe condition. Each model contained the time-series of the source region, the task regressors (safe and look), the interaction terms (PPIs) and the confound regressors (motion parameters, cue presentation, and ratings) (Fig. 1b). This procedure resulted in two different brain connectivity matrices for each run, consisting of contrast estimates for the safe and look condition. The connectivity matrices were then made symmetric by averaging corresponding (a-b, and b-a) parameter estimates, and were subsequently averaged across the four different runs. Subsequently the absolute values of the connectivity matrices were taken, and the connectivity matrices were additionally thresholded at 5%, (i.e., retaining only the strongest 5% of the connection).

To estimate the tonic brain connectivity an approach was followed as described by [15, 23]. For each time-series the task-related variance was “removed” by regressing a model containing task regressors and confounds (motion parameter, cue presentation, and ratings) on the time-series, and subsequently using the residual time-series for further analyses (Fig. 1b). The connectivity matrix was estimated by applying a graphical lasso [24] to the residuals of all nodes with the function graphicallasso.m (https://statweb.stanford.edu/~tibs/glasso) for a range of regularization parameters, lambda. The optimal regularization parameter lambda was estimated for each network (each run and each subject) by minimizing the Bayesian Information Criterion [25, 26]. These connectivity matrices were then averaged across the four different runs. The absolute value of the connectivity matrix was taken and entered in a graph theoretical analysis.

As a control to the connectivity analyses, the task-related activity per region was also estimated by a basic regression analyses of the task condition (safe and look, and safe > look) on the time-series of each region.

##### Functional module assignment

Each of the resulting brain nodes was assigned to a higher level functional module as proposed in [19]. The functional module assignment was performed as follows: the term related to each functional module was entered in Neurosynth [27], an automated meta-analysis tool; “Emotion”, “Motivation”, “Cognitive Control”, “Default Mode”, resulting in four (“association test”) images. The cortical region related to the term “emotion” that showed overlap with the cognitive control regions was assigned to the latter. Finally, the correspondence of the brain regions of the above-described parcellation and the four functional modules was estimated by testing the overlap of each region with any voxel of the resulting Neurosynth maps, see Fig. 4.

##### Network measures

The resulting phasic and tonic networks (absolute weighted graphs), where used to estimative several network measures [6], using the Brain Connectivity Toolbox (https://sites.google.com/site/bctnet/). For each node, the strength (the sum of connectivity values), local efficiency (average inverse shortest path length of a node), and the participation coefficient (diversity of intermodular connections of individual nodes) [28] were estimated (Fig. 1c). These measures were selected to capture some of the essential properties of network nodes (i.e., integration and segregation) which are suggested to be related to personality [29].

##### Classification: linear support vector machine

To estimate the predictive accuracy in classifying borderline personality disorder, a linear support vector machine (SVM) function implemented in Matlab (The MathWorks, Inc) was applied to the brain network measures and the task activity contrast estimates as a set of features. The advantage of using the network measures as features is that it yields a reduced set (here 121 features) of variables for classification, compared to using the element-wise connectivity (here 7260), and can thus also be regarded as a dimensionality reduction method while allowing inference on the brain node level.

The different network measures (strength, participation coefficient, local efficiency) for the phasic (safe > look, and the safe + look contrast) and tonic connectivity, as well as the main effect of the task (also safe > look, and the safe + look contrast) where entered as feature models (resulting in 11 different models). Before the classification procedure, the input feature data was “corrected” for the different sites by performing a regression analysis with dummy coded site regressors and then using the resulting residuals.

Each group was compared to each other group (one vs. one classification) in a nested-cross-validation procedure, where the balanced accuracy (the average of the sensitivity and specificity) served as the key outcome measure (effect size) of the classification performance. The data was divided in tenfold validation and test/train data. In the inner loop of the nested cross-validation, the test/train data was further divided in a fivefold cross-validation train and test data to estimate the balanced accuracy per model. The feature model with the highest balanced accuracy was then fitted to all test/training data of the inner loop, and the model was evaluated on the performance of the validation data of the outer loop [see Fig. 1 for an illustration of the procedure]. This process was repeated 200 times to obtain a stable estimate of the mean balanced accuracy and a 95% confidence interval.

The SVM classification was then repeated 1000 times with randomly permuted labels [25], to obtain a permutation null distribution. The p-value was defined as the number of times the null distribution showed a balanced accuracy higher than the average balanced accuracy of the validation data divided by the total number of permutations. Follow-up analyses were subsequently performed to aid the functional interpretation of the best performing model of the inner loop: (1) the SVM model weights were averaged across folds and repetitions resulting in an averaged model of feature weights, indicating the relative (by ranking [30]) contribution of each brain region in the classification (2) groups were tested on the difference of the network measures per functional module (emotion, motivation, cognitive control, default mode). The Matlab code for the network and SVM prediction analyses is available at https://github.com/henkcremers/NetworkAnalysis.

## Results

### Brain parcellation and subject inclusion

51 borderline patients, 26 cluster-C patients, and 44 non-patients were included in the current analyses, see Supplementary material 1 for details and Fig. S1.2 for the included brain nodes.

### Classification accuracy

**Fig. 2:**
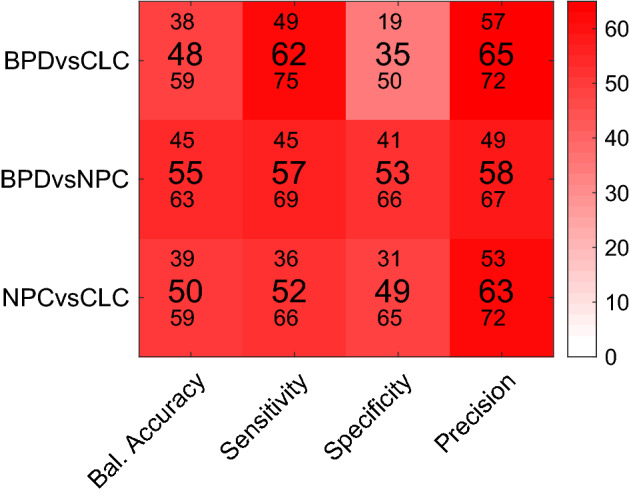
Performance of the SVM classification analysis. Each row represents the group comparison, and the columns indicate the outcome metrics. Values in the middle of each cell are the mean for a metric, the value above and below indicate the lower and upper bound of the 95% confidence interval. *Bal. Accuracy* balanced accuracy

The classification results for each group compared to each other group showed a limited cross-validated balanced accuracy, 55% CI95 [45 63] *p* = 0.23, for the BPD vs. NPC, 50% CI95 [39 59] *p* = 0.46 for the NPC vs. CLC, and 48%, CI95 [38 59], *p* > 0.5 for BPD vs. CLC, see Fig. 2 for an overview of these and other classification metrics. Thus, only the BPD vs. NPC classification showed a very modest, yet non-significant, effect.

### Exploratory analysis

**Fig. 3:**
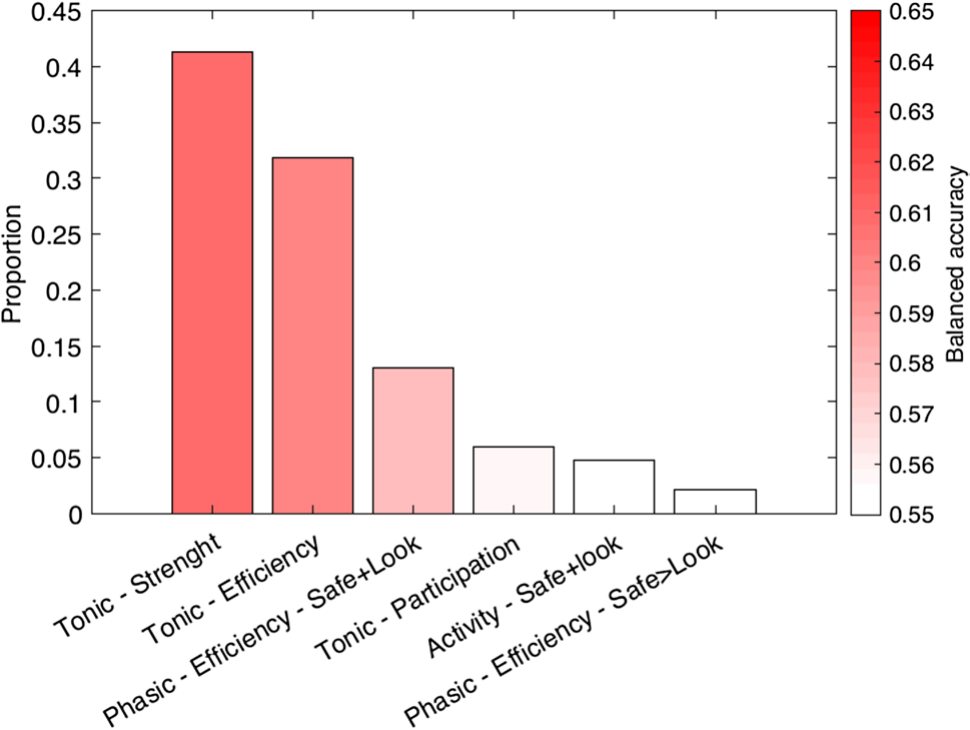
Results of the inner loop of the BPD vs. NPC model. The y-axis depicts the proportion of times the model was the highest performing model in the inner loop. The color-coding indicates the balanced accuracy of the inner loop of each model

**Fig. 4:**
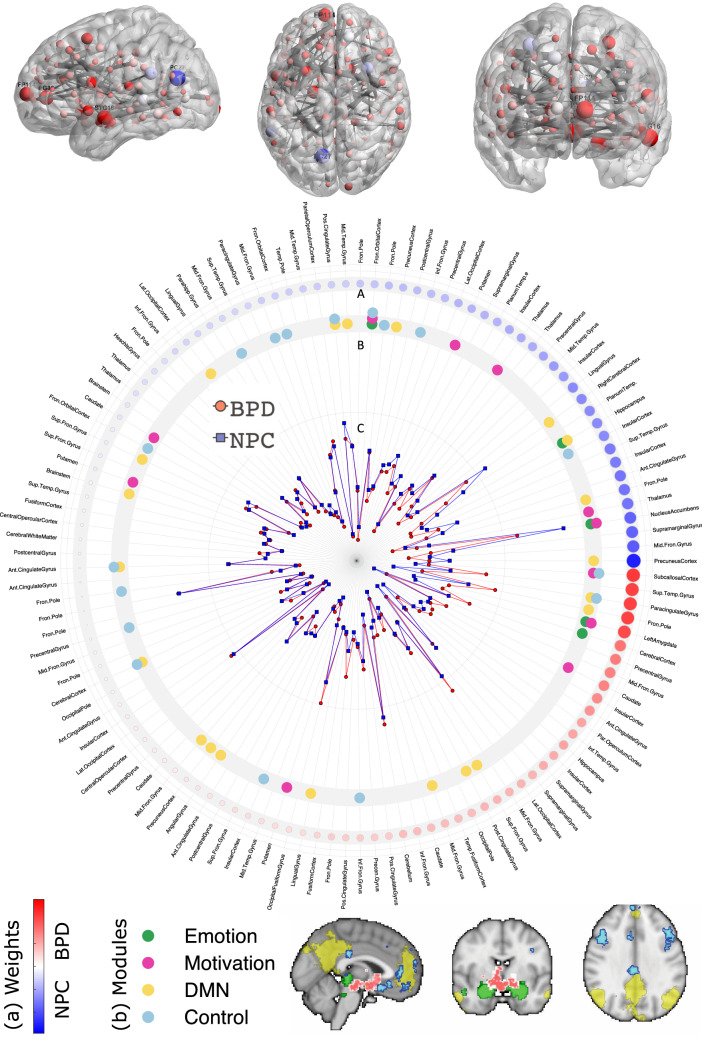
BPD vs. NPC Tonic-strength model. Upper panel the SVM weights plotted using BrainNet Viewer [31]. Red means a node has higher centrality in BPD, and blue represents higher centrality in NPC. The size of the nodes is proportional to the SVM weights. Middle panel from outside to inside: (A) rank-ordered averaged weights of the linear support vector machine, Color bar (bottom of figure), and node size indicates the relative contribution. (B) Functional module assignment, see bottom of figure for legend. (C) Node strength averaged per group, see lower panel for the legend. See supplementary Table S1 for a complete list of the nodes. Different nodes can contain the same label, see supplementary material

**Fig. 5:**
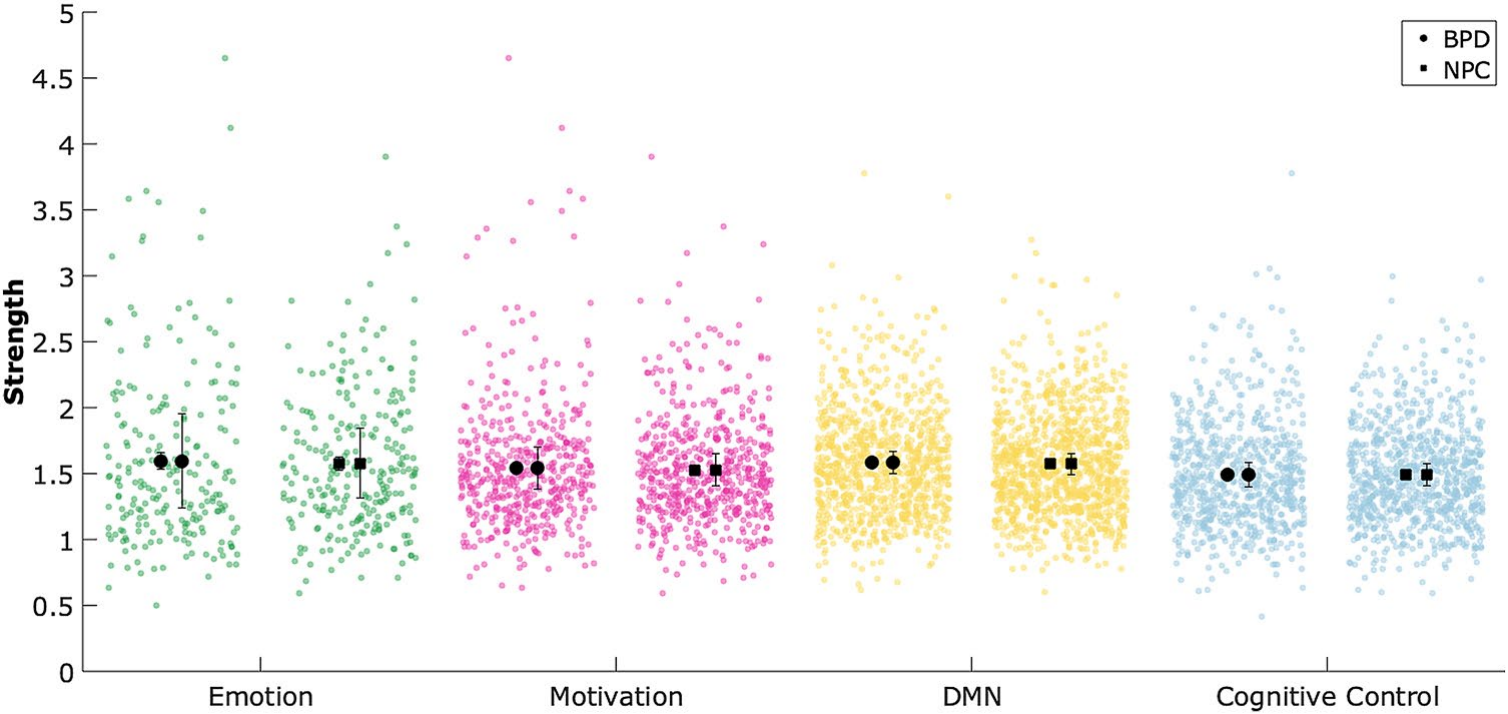
Manhattan style bar plot of strength centrality per module and group. The two adjacent black symbols per bar indicate the mean value of the subject per group (across regions; left of each pair) and mean of the different regions (across subjects; right of each pair)

We did opt to explore the results further to guide potential future research. Figure 3 shows the results of the balanced accuracy of the inner loop, which indicates that the *tonic strength* model was most often selected (40%), and showed a classification accuracy of 62%. Figure 4 then illustrates the rank-ordered features of the tonic strength BPD vs. NPC classification, the functional module assignment, and the averaged strength data per group. It is of note that the left amygdala was one of the 5 highest-ranking features and showed an increased strength (stronger connections) in the BPD group than the NPC group. The strength centrality was further tested on group differences between BPD and NPC for the four functional defined modules; emotion, motivation, cognitive control, and default mode. The results did not indicate any substantial difference (all Cohen’s *d* < 0.12, and all *p* > 0.5) between BPD and NPC (see Fig. 5).

## Discussion

The current study used network measures in a machine learning approach to classify BPD patients when engaged in an emotion regulation task. There was a small, yet not statistically significant, classification accuracy for BPD vs. NPC of 55%. Within the nested classification procedure, the tonic strength model showed the highest balanced classification accuracy of 62%. Contrary to our expectations, the network measure models based on phasic connectivity performed worse in classifying BPD vs. NPC. While statistical significance testing is arbitrary and heavily debated [32, 33], the effects found in our study (classification accuracy) are undoubtedly small. First, we will consider possible explanations for this and recommendations for future research; then we will evaluate the potential of the analytic procedure.

The current data set is the largest BPD study [18] in the published literature [3, 34]. At the same time, the sample size is still modest considering statistical power in general [35], and arguably even more so for machine learning analyses [36]. On the one hand, machine learning analyses focuses on the classification performance on an entire model and hence consist of a single (or a few) outcome measures which improve power over mass-univariate testing [35]. However, there is a large error surrounding the cross-validation accuracy in relatively small samples [36]. Indeed, the estimated confidence intervals here (± 8–10%) indicate a large variability of the balanced accuracy, and the permutation distributions showed a large standard deviation. Moreover, in the nested cross-validation, only a subset of the data is used for model testing, and we did observe a substantial drop in balanced accuracy from the inner to the outer loop (see [11] for a discussion). The subsampling analyses did display an increase in balanced accuracy as a function of sample size for the BPD vs. NPC classification (see supplementary material 3), showing that larger samples could lead to increased classification accuracy. In sum, these limitations underscore the necessity to further study this topic, preferably in a larger (for example, aggregated) sample.

Previous machine learning fMRI research on BPD [12, 37, 38] did show a substantially higher classification accuracy, yet there are some methodological factors to consider. While machine learning approaches, through cross-validation, provide an estimate of the generalization of a model [35], they are nonetheless also susceptible to methodological variability and researcher degrees of freedom [39]. Furthermore, the cross-validation procedures used in other studies (leave-one-out cross-validation) tend to overestimate accuracy [11]. Lastly, previous BPD classification studies contained much smaller sample sizes, which, in combination with a certain degree of flexibility in analytic strategies can lead to possibly inflated effect sizes [35] and the discussed large confidence intervals surrounding the estimates. In general, an overview of fMRI based classification of psychiatric disorders showed that when tested on validation/external data, classification accuracy drops substantially [40].

Aside from the above-mentioned methodological issues, there are critical clinical and biological aspects to deliberate when evaluating the balanced classification accuracy. BPD is, like most psychiatric disorders, a heterogeneous construct: patients with different symptom profiles can be diagnosed with BPD. In addition, the BPD diagnostic criteria do not incorporate any brain markers—for good reasons, since no reliable biomarker has been identified yet [41]. Clinical and biological heterogeneity of BPD imposes a low ceiling on the maximum classification accuracy one can realistically expect. That is, while in principle, a heterogeneous clinical construct could have a homogenous biological basis (i.e., multifinality [42]), we regard this to be unlikely in BPD. A potential future research avenue is to test if clinically identified subtypes of BPD [43] are also potentially more biologically homogenous in terms of brain network organization. In this case, classifying these subtypes with network centrality might be possible with much higher accuracy.

While the overall classification accuracy thus was small, we did further explore the highest performing model (tonic strength) to indicate the possibilities of network measures as features in classification analyses. As noted in the method section, a benefit of network measures is the dimensionality reduction it involves and making inferences possible at the node level. For example, the amygdala was one of the five highest-ranking positive features in the classification of BPD vs. NPC. The amygdala is considered to be a critical region in almost any form of psychopathology, including BPD [3]. The BPD patients displayed higher amygdala connectivity strength than the NPC. This indicates that the amygdala in BPD is more interconnected with the rest of the brain, which could signal an increase in arousal [44] to other brain regions. At the same time, the follow-up analyses of the strength centrality of regions in the functional modules (emotion, motivation, cognitive control, and default mode network), did not show any substantial difference between the BPD and NPC. This could imply that there is no particular functional network that differentiates BPD from NPC, and that distributed and subtle differences across brain regions represent the neural mechanism underlying BPD. However, it is also possible that there is considerable variation among individual subjects concerning which cognitive control regions are most involved in this emotion regulation task, which would also create this observed scattered pattern.

Contrary to our expectations, as mentioned, we found that the phasic connectivity (connectivity that depends on specific task-events) models were not able to detect BPD vs. NPC. Psychophysiological interaction analysis is a well-established approach to assess phasic task-related connectivity. However, a principal shortcoming of this analysis (and more generally, the testing of interaction effects [45]) is that by controlling for the “main” effects of task and time-series, the psychophysiological interaction term needs to demonstrate a large effect above and beyond these main effects [22]. It is unknown if this could explain the limited success in classifying BPD vs. NPC based on the phasic connectivity, but it is a principle difficulty in assessing phasic connectivity changes. Also, here, more research is needed to evaluate the predictive power of phasic connectivity in classifying BPD patients and other psychiatric disorders. In that sense, this discussion can be regarded as part of a broader discussion on the added benefit of investigating dynamic vs. stationary connectivity [46, 47].

A fundamental limitation is the difference between the participating sites in the angle (tilt) of the field of view in fMRI data acquisition, and consequently, the loss of data. We have addressed this problem by performing a trade-off analysis of minimal node intensity as an index of data quality and subject inclusion yet aiming to incorporate important subcortical and cortical nodes. Nonetheless, seven subjects were excluded from the current analyses, and a part of the brain was not included in the analyses. This might have limited the classification accuracy, yet to the best of our knowledge, not biased the results.

## Conclusion

The current study found a small and non-significant effect of BPD classification based on the brain network measures. Larger samples are needed to more accurately assess how well BPD patients can be classified. Future research should, furthermore, focus on clinically or biologically defined potential subtypes of BPD. Finally, a better understanding of the neural network organization and connectivity of borderline personality disorder could eventually have the potential to optimize individually tailored treatments.

## Electronic supplementary material

Below is the link to the electronic supplementary material.

Supplementary file1 (DOCX 435 kb)
