# Supplementary material for: Borderline personality disorder classification based on brain network measures during emotion regulation
Source: Eur Arch Psychiatry Clin Neurosci. 2020 Dec 2;271(6):1169–78. doi: 10.1007/s00406-020-01201-3 (PMC8354902; doi:10.1007/s00406-020-01201-3)
Supplement: Supplementary file 1 — Supplementary file1 (DOCX 435 kb) [file 406_2020_1201_MOESM1_ESM.docx]

**Borderline Personality Disorder classification based on brain network measures during emotion regulation**

**Supplementary Material**

S1. Brain parcellation.

S2. Phasic and Tonic network properties

S3. Effect of subsampling.

**S1. Brain parcellation and subject inclusion**

Brain Parcellation

The brain parcellation was based on the spatially constrained spectral cluster method [1], as applied in Cremers et al., 2017; https://osf.io/ds5jx/), and originally consisted of 219 nodes. For these initial 219 nodes the average time-series was extracted for each subject. Due to differences in the tilt of the field of view during the data acquisition, the functional coverage differed across sites and subjects. To maintain a minimum signal intensity of regions across all included subjects (as a proxy for the data quality), we opted to perform a trade-off analysis of subject and node inclusion/exclusion for a range of minimal signal intensities (defined as the fraction of the mean signal intensity), see figure S1. Based on the visual inspection of the node inclusion for different intensity threshold (we aimed for a large subcortical coverage), and preferential minimal subject dropout, we opted for a minimal signal intensity of 30% of the mean for each subject, and a maximum exclusion of 5% of the subjects. This resulted in the inclusion of 121 nodes (including coverage of the amygdala), see figure S2. 7 subjects as compared to Ref [2] (5 borderline patients, 1 non-patient, 1 Cluster-C patient) were excluded from further analyses resulting in 51 borderline patients, 26 cluster-C patients and 44 non-patients.

**Figure S1.1.** Trade-off between region and subject dropout for a range of minimal signal intensity (fraction of mean intensity, see legend).

**
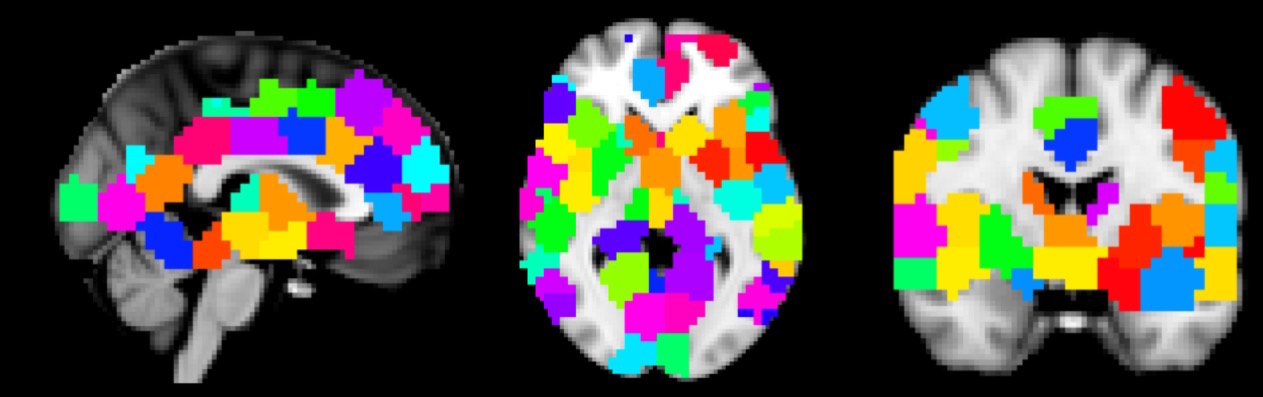
**

**Figure S1.2**. Resulting 121 brain regions utilized in the network analysis.

**Table S1.1** List of all included brain regions. Note that all labels are based on the Harvard-Oxford atlas as implemented in FSL [3], and different nodes can contain the same label.

| **x** | **y** | **z** | **k** | **NR** | **Name** |
| --- | --- | --- | --- | --- | --- |
| 11 | -31 | 43 | 568 | **1** | Cingulate Gyrus, posterior division |
| -15 | -30 | -20 | 736 | **2** | Brainstem |
| 45 | 30 | -11 | 624 | **3** | Frontal Orbital Cortex |
| 13 | 22 | 58 | 568 | **4** | Superior Frontal Gyrus |
| 51 | -67 | 5 | 624 | **5** | Lateral Occipital Cortex |
| 55 | 12 | 15 | 664 | **6** | Inferior Frontal Gyrus |
| -2 | 45 | 37 | 696 | **7** | Superior Frontal Gyrus |
| 23 | -70 | -13 | 936 | **8** | Occipital Fusiform Gyrus |
| -7 | -31 | 43 | 768 | **9** | Cingulate Gyrus, posterior division |
| -13 | -54 | -1 | 896 | **10** | Lingual Gyrus |
| -33 | 19 | 1 | 688 | **11** | Insular Cortex |
| -44 | 31 | -10 | 616 | **12** | Frontal Orbital Cortex |
| 5 | -35 | -14 | 552 | **13** | Brainstem |
| 45 | 1 | 48 | 737 | **14** | Precentral Gyrus |
| 46 | -28 | 16 | 672 | **15** | Parietal Operculum Cortex |
| -54 | -4 | -13 | 640 | **16** | Superior Temporal Gyrus, anterior division |
| 8 | 43 | 2 | 704 | **17** | Cingulate Gyrus, anterior division |
| -12 | 4 | 13 | 320 | **18** | Caudate |
| -43 | -33 | 14 | 712 | **19** | Planum Temporale |
| -27 | 57 | 2 | 512 | **20** | Frontal Pole |
| 14 | -95 | 0 | 710 | **21** | Occipital Pole |
| -30 | 37 | 32 | 642 | **22** | Middle Frontal Gyrus |
| 12 | -21 | 7 | 656 | **23** | Thalamus |
| -40 | 20 | 39 | 615 | **24** | Middle Frontal Gyrus |
| -26 | 26 | 45 | 591 | **25** | Middle Frontal Gyrus |
| -56 | -9 | 28 | 624 | **26** | Postcentral Gyrus |
| -10 | -67 | 21 | 896 | **27** | Precuneus Cortex |
| -13 | 14 | -3 | 640 | **28** | Caudate |
| 28 | 0 | -1 | 800 | **29** | Putamen |
| 45 | 9 | 31 | 784 | **30** | Precentral Gyrus |
| 5 | 10 | 46 | 720 | **31** | Paracingulate Gyrus |
| -56 | -26 | -7 | 752 | **32** | Middle Temporal Gyrus, posterior division |
| 9 | -74 | 4 | 1104 | **33** | Cerebral White Matter |
| 63 | -19 | 10 | 926 | **34** | Planum Temporale |
| 14 | -2 | 15 | 288 | **35** | Caudate |
| -6 | 46 | 4 | 672 | **36** | Paracingulate Gyrus |
| 44 | 44 | 10 | 634 | **37** | Frontal Pole |
| -48 | -62 | 23 | 775 | **38** | Lateral Occipital Cortex, superior division |
| 53 | -55 | 21 | 884 | **39** | Angular Gyrus |
| 42 | -8 | -8 | 832 | **40** | Insular Cortex |
| 2 | 20 | 30 | 704 | **41** | Cingulate Gyrus, anterior division |
| -25 | 11 | 55 | 752 | **42** | Middle Frontal Gyrus |
| -44 | 7 | 31 | 720 | **43** | Precentral Gyrus |
| -13 | -32 | 0 | 704 | **44** | Thalamus |
| 15 | -65 | 23 | 832 | **45** | Precuneus Cortex |
| -37 | -15 | -3 | 696 | **46** | Insular Cortex |
| -42 | 41 | 11 | 656 | **47** | Frontal Pole |
| 2 | -17 | 33 | 512 | **48** | Cingulate Gyrus, posterior division |
| -57 | -50 | -10 | 656 | **49** | Middle Temporal Gyrus, temporo-occipital part |
| -25 | 0 | 0 | 736 | **50** | Putamen |
| 37 | 19 | 1 | 816 | **51** | Insular Cortex |
| -17 | 53 | 28 | 536 | **52** | Frontal Pole |
| -10 | -73 | 4 | 832 | **53** | Lingual Gyrus |
| -42 | 0 | 47 | 799 | **54** | Precentral Gyrus |
| 2 | -5 | 4 | 608 | **55** | Thalamus |
| 2 | -53 | 14 | 832 | **56** | Precuneus Cortex |
| -8 | -19 | 9 | 632 | **57** | Thalamus |
| -38 | 0 | 7 | 576 | **58** | Insular Cortex |
| 42 | 24 | 38 | 719 | **59** | Middle Frontal Gyrus |
| -45 | 26 | 23 | 664 | **60** | Inferior Frontal Gyrus, pars triangularis |
| 53 | -13 | 40 | 679 | **61** | Postcentral Gyrus |
| -55 | -55 | 8 | 792 | **62** | Middle Temporal Gyrus, temporo-occipital part |
| -28 | -37 | -13 | 784 | **63** | Parahippocampal Gyrus, posterior division |
| -3 | 33 | 20 | 632 | **64** | Cingulate Gyrus, anterior division |
| -58 | -25 | 14 | 696 | **65** | Parietal Operculum Cortex |
| 16 | 13 | 7 | 416 | **66** | Caudate |
| 53 | -29 | -2 | 704 | **67** | Superior Temporal Gyrus, posterior division |
| 22 | -11 | -17 | 784 | **68** | Hippocampus |
| 1 | 28 | 45 | 824 | **69** | Superior Frontal Gyrus |
| 41 | 0 | 8 | 608 | **70** | Insular Cortex |
| 2 | -20 | -9 | 728 | **71** | Thalamus |
| 62 | -39 | 23 | 654 | **72** | Supramarginal Gyrus, posterior division |
| 14 | 43 | 44 | 592 | **73** | Frontal Pole |
| -46 | -72 | 9 | 704 | **74** | Lateral Occipital Cortex, inferior division |
| 19 | -56 | 2 | 952 | **75** | Lingual Gyrus |
| 34 | -31 | -17 | 760 | **76** | Temporal Fusiform Cortex, posterior division |
| 60 | -3 | 27 | 717 | **77** | Precentral Gyrus |
| -49 | 22 | 7 | 584 | **78** | Inferior Frontal Gyrus, pars triangularis |
| 12 | 62 | 8 | 496 | **79** | Right Cerebral Cortex |
| 28 | 31 | 44 | 711 | **80** | Middle Frontal Gyrus |
| 0 | 15 | -9 | 4 | **81** | Subcallosal Cortex |
| -1 | 4 | 35 | 560 | **82** | Cingulate Gyrus, anterior division |
| -5 | -93 | 4 | 640 | **83** | Occipital Pole |
| -18 | -8 | -17 | 776 | **84** | Left Amygdala |
| -53 | 7 | 17 | 616 | **85** | Precentral Gyrus |
| 60 | -46 | 7 | 903 | **86** | Middle Temporal Gyrus |
| 31 | 14 | 53 | 685 | **87** | Middle Frontal Gyrus |
| 3 | -53 | -11 | 784 | **88** | Cerebellum |
| -27 | 13 | -18 | 720 | **89** | Frontal Orbital Cortex |
| 51 | 11 | -6 | 672 | **90** | Temporal Pole |
| -57 | -44 | 25 | 744 | **91** | Supramarginal Gyrus, posterior division |
| 0 | 54 | 21 | 608 | **92** | Cerebral Cortex |
| 32 | 43 | 28 | 706 | **93** | Frontal Pole |
| -47 | -67 | -8 | 784 | **94** | Lateral Occipital Cortex, inferior division |
| 20 | -35 | -3 | 688 | **95** | Hippocampus |
| -49 | -18 | 42 | 576 | **96** | Postcentral Gyrus |
| -47 | 10 | -7 | 816 | **97** | Insular Cortex |
| 59 | -9 | -11 | 6 | **98** | Superior Temporal Gyrus, posterior division |
| 49 | 28 | 22 | 717 | **99** | Middle Frontal Gyrus |
| 54 | -59 | -8 | 689 | **100** | Inferior Temporal Gyrus, temporo-occipital part |
| -55 | -9 | 6 | 744 | **101** | Central Opercular Cortex |
| 9 | 36 | 23 | 536 | **102** | Cingulate Gyrus, anterior division |
| 15 | 56 | 27 | 575 | **103** | Frontal Pole |
| -26 | -56 | -14 | 856 | **104** | Temporal Occipital Fusiform Cortex |
| -13 | 34 | 50 | 600 | **105** | Superior Frontal Gyrus |
| -59 | -38 | 2 | 784 | **106** | Superior Temporal Gyrus, posterior division |
| 41 | -16 | 9 | 536 | **107** | Heschls Gyrus |
| 15 | 13 | -7 | 608 | **108** | Putamen |
| 31 | 54 | 14 | 5 | **109** | Frontal Pole |
| 29 | -51 | -15 | 904 | **110** | Temporal Occipital Fusiform Cortex |
| -37 | -1 | -16 | 6 | **111** | Cerebral Cortex |
| 3 | -9 | 47 | 824 | **112** | Cingulate Gyrus, anterior division |
| 52 | 30 | 4 | 543 | **113** | Inferior Frontal Gyrus, pars triangularis |
| -8 | 63 | 7 | 456 | **114** | Frontal Pole |
| -56 | -29 | 34 | 656 | **115** | Supramarginal Gyrus, anterior division |
| -29 | 51 | 18 | 607 | **116** | Frontal Pole |
| 58 | -2 | 4 | 712 | **117** | Central Opercular Cortex |
| 2 | -38 | 30 | 632 | **118** | Cingulate Gyrus, posterior division |
| -39 | -17 | 12 | 520 | **119** | Insular Cortex |
| 61 | -25 | 29 | 611 | **120** | Supramarginal Gyrus, anterior division |
| 1 | -4 | -11 | 632 | **121** | Nucleus Accumbens |

**Table S1.2** Sample characteristics of the three groups: borderline personality disorder (BPD), non-patient controls (NPC), and cluster-C control patients (CCP).

|  | BPD | NPC | CCP | Test Statistics | |
| --- | --- | --- | --- | --- | --- |
|  | (*n* = 51) | (*n* = 44) | (*n* = 26) | *F* | *p* |
| Age, years, mean (SD) | 31.00 (8.98) | 28.84 (10.85) | 29.96 (10.45) | 0.55 | .579 |
| Education level^a^, No. (%) |  |  |  | 13.95 | .001^b^ |
| Level 1 | 14 (27.5) | 7 (15.9) | 5 (19.2) |  |  |
| Level 2 | 9 (17.6) | 2 (4.5) | 3 (11.5) |  |  |
| Level 3 | 13 (25.5) | 3 (6.8) | 6 (23.1) |  |  |
| Level 4 | 2 (3.9) | 2 (4.5) | 4 (15.4) |  |  |
| Level 5 | 11 (21.6) | 20 (45.5) | 4 (15.4) |  |  |
| Level 6 | 2 (3.9) | 9 (20.5) | 4 (15.4) |  |  |
| Estimated IQ^c^, mean (SD) | 96.55 (11.21) | 101.91(10.96) | 95.42 (12.39) | 3.60 | .030 |
| Handedness, No. L/R/M | 5/43/2 | 2/42/- | -/25/1 | 5.17 | .270 ^d^ |
| BSI, mean (SD), total | 1.77 (.55) | 0.18 (.24) | 1.20 (.55) | 136.59 | < .001^e^ |
| BPD checklist, mean (SD), total | 123.22 (26.01) | 52.83 (8.69) | 81.80 (25.30) | 127.47 | < .001^f^ |
| ITEC, mean (SD) | 69.09 (32.61) | 6.13 (8.38) | 33.87 (26.26) | 64.39 | < .001^g^ |
| Sexual abuse | 9.12 (8.61) | 0.08 (0.32) | 2.12 (4.92) | 24.50 | < .001 |
| Physical abuse | 17.57 (11.26) | 1.68 (3.50) | 7.55 (11.13) | 31.08 | < .001 |
| Emotional abuse | 20.38 (8.74) | 2.77 (4.41) | 12.58 (8.18) | 58.46 | < .001 |
| Emotional neglect | 11.36 (6.82) | 0.91 (2.05) | 7.51 (7.47) | 33.04 | < .001 |
| Physical neglect | 10.66 (9.28) | 0.69 (2.50) | 4.11 (5.91) | 22.45 | < .001 |
| Clinical disorders, No. (%) |  |  |  |  | *p*^h^ |
| Major depressive disorder | 45 (88.2) |  | 16 (61.5) | 7.46 | .006 |
| Dysthymic | 4 (7.8) |  | 4 (15.4) | 1.05 | .305 |
| Bipolar type II | 1 (2.0) |  | - | 0.52 | .472 |
| Generalized anxiety disorder | 1 (2.0) |  | 1 (3.8) | 0.24 | .623 |
| Panic disorder with agoraphobia | 6 (11.8) |  | 2 (7.7) | 0.31 | .580 |
| Panic disorder | 7 (13.7) |  | 2 (7.7) | 0.61 | .436 |
| Agoraphobia | 4 (7.8) |  | - | 2.15 | .142 |
| Specific phobia | 8 (15.7) |  | - | 4.55 | .033 |
| Social phobia | 18 (35.3) |  | 5 (19.2) | 2.12 | .145 |
| Obsessive compulsive disorder | 7 (13.7) |  | 2 (7.7) | 0.61 | .436 |
| Posttraumatic stress disorder | 19 (37.3) |  | 3 (11.5) | 5.58 | .018 |
| Somatoform disorder | 5 (9.8) |  | 4 (15.4) | 0.52 | .471 |
| Eating disorders | 19 (37.3) |  | 10 (38.5) | 0.01 | .981 |
| Substance abuse | 27 (52.9) |  | 2 (7.7) | 15.02 | < .001 |
| Personality disorders, No. (%) |  |  |  |  |  |
| Avoidant PD | 24 (47.1) |  | 19 (73.1) | 4.73 | .030 |
| Dependent PD | 9 (17.6) |  | 4 (15.4) | 0.06 | .802 |
| Obsessive compulsive PD | 9 (17.6) |  | 8 (30.8) | 1.72 | .189 |
| Passive aggressive PD | 3 (5.9) |  | 1 (3.8) | 0.15 | .703 |
| Depressive PD | 13 (25.5) |  | 2 (7.7) | 3.48 | .062 |
| Paranoid PD | 15 (29.4) |  | 1 (3.8) | 6.84 | .009 |
| Schizotypal PD | 1 (2.0) |  | - | 0.52 | .472 |
| Schizoid PD | 1 (2.0) |  | 1 (3.8) | 0.24 | .623 |
| Medication, No. (%) |  |  |  |  |  |
| Antidepressants | 34 (66.7) |  | 8 (30.8) | 8.95 | .003 |
| Antipsychotics | 8 (15.7) |  | - | 4.55 | .033 |
| Hypnotics | 2 (3.9) |  | - | 1.05 | .306 |
| Mood Stabilizers | 1 (2.0) |  | - | 0.52 | .472 |

L = Left; R = Right; M = Mixed; BSI = Brief Symptom Inventory; BPD checklist = Borderline checklist; ITEC = Interview Traumatic Events Childhood; PD = Personality Disorder.

^a^ Level of education of both the Dutch and German educational systems were translated into the International Standard Classification of Education (ISCED), in current study six levels of education were divided ranging from lower secondary school to Master’s degree.

^b^ Value is based on Kruskal-Wallis, data of one NPC not available.

^c^ Assessed with four subtasks of the WAIS, data of one NPC not available.

^d^ Value is based on Chi-square, data of one BPD patient not available.

^e^ All three groups significantly differed from each other (*p* < .001), data of two NPC not available.

^f^ All three groups significantly differed from each other (*p* < .001), data of two NPC and one CCP not available.

^g^ MANOVA and ANOVAs showed significant group effects over traumas. BPD patients experienced significantly more trauma compared to both control groups regarding sexual abuse (vs. NPC *p* < .001; vs. CCP *p* < .001), physical abuse (vs. NPC *p* < .001; vs. CCP *p* < .001) and physical neglect (vs. NPC *p* < .001; vs. CCP *p* = .001). The three groups significantly differed from each other concerning emotional abuse (BPD vs. NPC *p* < .001; BPD vs. CCP *p* = .003; NPC vs. CCP *p* < .001) and emotional neglect (BPD vs. NPC *p* < .001; BPD vs. CCP *p* = .023; NPC vs. CCP *p* < .001), with BPD patients experiencing the most trauma, followed by the CCP and the NPC experienced the least trauma. Data of eight NPC and one CCP not available.

^h^ Value is based on Chi-square.

As mentioned, the subject inclusion for the current analysis is not identical to the previous report [2] bases on this study, resulting in small differences with respect to demographics variables.

**S2. Network Properties.**

Overview of the basic network properties. Figure S2 shows the distribution of the strength centrality, and the table show the global network properties: clustering coefficient, Modularity, Efficiency and Strength.

**Figure S2 – strength distribution**

**Table S2. Main network properties.**

Clustering Coefficient, Modularity, Global Efficiency, and the average absolute strength, per group.

**S3. Effect of subsampling**

To assess the stability of the classification results and test for a potential trend between sample size and the cross-validated balanced accuracy the support vector machine procedure was repeated for 100 random subsamples for a range of sample sizes (50% - 100% of the data), see figure S3a. The fitted power curve for the BPD vs NPC classification was then used to project the balanced accuracy for a larger range of samples, see figure 3b. Do note that this projection is highly uncertain, and merely used to give an indication of the benefit of larger samples.

**Figure S3a.** Relation between subsampling and balanced accuracy. Sample size (x-axis) and cross-validated balanced accuracy (averaged over 100 repetitions of random subsamples). The solid red line indicates the fitted power curve, and dashed lines the prediction bounds.


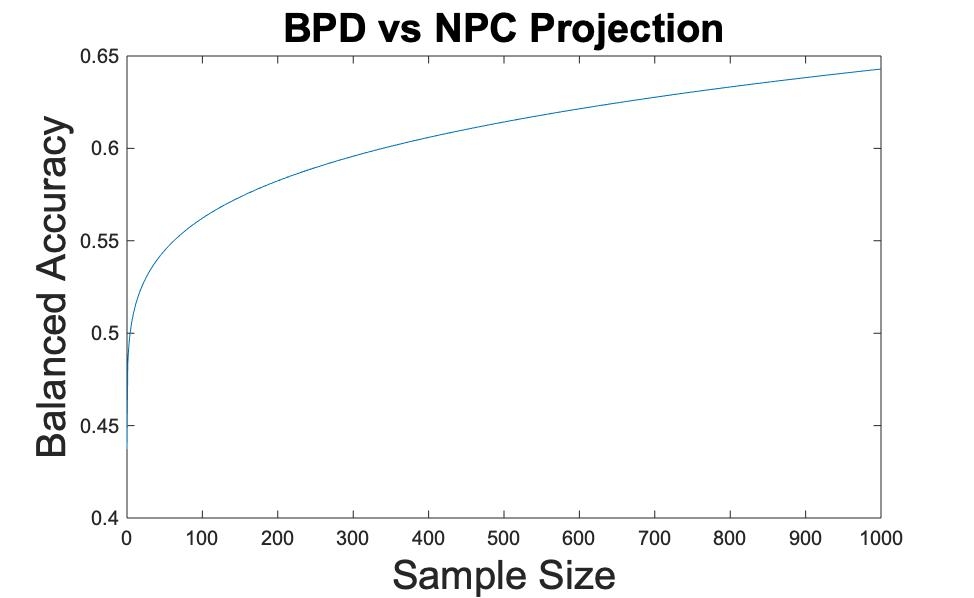


**Figure S3b.** Projected balanced accuracy for BPD vs NPC as a function of sample size.

**References**

1. Craddock RC, James GA, Holtzheimer PE, et al (2012) A whole brain fMRI atlas generated via spatially constrained spectral clustering. Hum Brain Mapp 33:1914–1928. doi: 10.1002/hbm.21333

2. van Zutphen L, Siep N, Jacob GA, et al (2017) Always on guard: emotion regulation in women with borderline personality disorder compared to nonpatient controls and patients with cluster-C personality disorder. J Psychiatry Neurosci 43:170008–47. doi: 10.1503/jpn.170008

3. Desikan RS, Ségonne F, Fischl B, et al (2006) An automated labeling system for subdividing the human cerebral cortex on MRI scans into gyral based regions of interest. NeuroImage 31:968–980. doi: 10.1016/j.neuroimage.2006.01.021
